# Optic Neuritis and Its Significance as a Symptom1Read before the Central New York Medical Association, November 19, 1889.

**Published:** 1890-02

**Authors:** Alvin A. Hubbell

**Affiliations:** Professor of Ophthalmology, Otology, and Laryngology in the Medical Department of Niagara University; 212 Franklin Street


### OPTIC NEURITIS AND ITS SIGNIFICANCE AS A SYMPTOM.[1]

By ALVIN A. HUBBELL, M. D.,

Professor of Ophthalmology, Otology, and Laryngology in the Medical Department of Niagara University.

THE announcement of the subject "Optic Neuritis," may carry with it the impression that I am about to discuss a theme which belongs preëminently to ophthalmology, and has very little in it of interest to one who is not specially engaged in ophthalmic practice. But permit me to assure you that such is not the case, for its consideration is of greatest value to the physician, and it has a significance to him of peculiar and deep meaning in the investigation, diagnosis, and treatment of a certain class of diseases. It is only incidentally that the ophthalmic surgeon should have anything to do with this condition, and then it should only be in association with the physician, who accepts his aid because his more frequent use of the ophthalmoscope has, perhaps, trained him to quicker and more accurate appreciation of changes found in the fundus of the eye. Optic neuritis is but a symptom of disease which the physician alone is called up to study and treat; and, because of this, he, above all others, should be able to recognize it, to intelligently study it, and to interpret its meaning, notwithstanding it requires some knowledge of ophthalmoscopy to do so.

And here let me parenthetically remark that the lack, on the part of the physician of to-day, of the knowledge of the use of the ophthalmoscope, and of the ability to use it, puts him behind the times, and cripples him in his efforts to arrive at correct diagnoses and apply such proper treatment as only well-grounded conclusions can suggest. He who fails to use this instrument in his every-day practice lays aside one which is of greatest diagnostic value, (and, withal, easy to use,) and the appearances which it presents are easy to recognize and understand. I trust the time is not far distant when every medical school will require of its graduates a practical knowledge of its workings.

Optic neuritis, otherwise known as "papillitis," "choked disc," "edema of the disc," etc., is an affection of the optic nerve in some portion of its extent characterized by inflammatory action, edema, or degenerative changes, or all of these combined, and the results which attend or follow each or all of these processes.

### ANATOMICAL CONSIDERATIONS.

To bring out more clearly some of the pathological aspects of this disease, let us pause a moment to recall some anatomical points in

1. Read before the Central New York Medical Association, November 19, 1889.

regard to the optic nerve and its relations. Arising from the optic commissure at the sella turcica, it passes forwards through the optic foramen into the orbit to the eyeball, and joins the latter at a point to the inner side of its posterior pole. On leaving the cranial cavity it carries with it, and becomes invested throughout its orbital course by the same membranes which surround the brain, and these enclose spaces which correspond to and communicate with the subdural and subarachnoidal spaces of the brain. The pia mater becomes the inner sheath, and the dura mater the outer sheath of the optic nerve, and the space between them, the inter-vaginal space which is more or less completely subdivided into two spaces by the arachnoid membrane. Besides communicating with the brain-cavities, this space also communicates with "lymph" spaces within the optic nerve, and with those surrounding the retinal vessels in the eye. The optic nerve becomes very much constricted at its junction with the ball, its sheaths unite with the sclerotic, and its fibers, divested of their coverings, pass into the interior of the eye through the lamina cribrosa and choroidal opening, and bending at an acute angle expand to form the nerve-fiber layer of the retina. Posteriorly to the ball, the optic nerve-fibers are held together in bundles by connective-tissue, a slight amount of which is also carried into the eye, becoming, here, transparent. The retinal vessels are transmitted through the bulbar end of the optic nerve and enter the eye near the center of its intraocular extremity, known as the optic disc. In the eye, they at first pass upwards and downwards over the disc by one or two primary branches, and these soon divide and subdivide into smaller branches, which ramify in the retina over every part of the fundus, except at the macula lutea.

### NORMAL APPEARANCES.

The optic disc is round in shape, and is readily recognized by the contrast which its lighter color gives as compared with the bright, red, choroidal reflex from the rest of the fundus. Its center is usually whitish in color, but beyond this and toward its periphery, it has a reddish or rosy tint. Its surface is nearly flat, with a slight depression at the center, its edges or outlines are clear and well-defined, and the retinal vessels emerging from near the center pass over it in almost a straight course, while over the retina they take long curves. By the direct method of using the ophthalmoscope, the larger vessels reflect a light-colored streak or line along their length.

The intraocular portion of the optic nerve is the only part of it that can be seen, and bearing in mind the normal appearances briefly noted above, we can appreciate some of those changes which mark the progress of an optic neuritis, and which the ophthalmoscope presents.

### APPEARANCES OF OPTIC NEURITIS.

This disease, like all others, may be so slight as hardly to be recognized, or it may be so severe as to make its recognition unmistakable, even to the inexperienced. Its degree of intensity may not only vary, but it may assume different appearances by variations in the character of the process. Thus, the inflammation may be largely interstitial, affecting the connective tissue or neurilemma of the nerve-stem most, when the optic disc will show less deviation from the normal. Or the inflammation may partake more of the edematous process, when the disc will show a more or less pronounced and exaggerated change. Again, if seen late in its progress, degenerative action may have taken place, when still another appearance is presented.

Thus the picture of optic neuritis varies according to its degree, type, and stage. In its active stage, the essential characteristics which are to be looked for and relied upon, are: *First*, Change of color of the optic disc. This is first from the rosy tint to red, and then to a reddish-grey, or lilac-grey, with fine, radiating, red, and grey striations extending around the now indistinct border and over its surface. This has been significantly called "wooly." *Second*, Obscuration of the edges of the disc. In mild cases, and in the early stages, this is first seen where the optic-nerve fibers are most numerous, that is, above, below, and at the nasal side. A slight obscuration is best seen by the direct method of using the ophthalmoscope. If any doubt exists as to whether or not the appearance of blurring is abnormal, comparison should be made with the disc of the other eye, as the neuritis, if present, may be more intense in one than in the other, and the more pronounced obscurity of the edges of one makes the presence of the disease more certain in both. As the case progresses in severity, the whole circumference becomes obscured and fringed with the radiating striations of grey and red, above described; or it may merge in color with the surrounding fundus, no trace of outline being left. *Third*, Swelling of the optic disc. This swelling causes the disc to appear enlarged and increased in height. The extent of enlargement is often two or three times the diameter of the normal disc, and the height sometimes reaches two or three millimeters. The increased size is easily recognized, and the height is measured with the ophthalmoscope in the same way as one would measure refraction of the eye, the difference between the highest point of the swelling and the level of the surrounding retina determining the degree, allowing about one millimeter for every three dioptries of difference shown by the ophthalmoscope. The height is also indicated by the extent of parallactic movement, or apparent change in position, which it undergoes in relation to structures on different levels when the head of the observer by the direct method, or the object lens by the indirect method, is moved up or down or from side to side. Again, the prominence is shown by the appearance of the vessels, when these can be seen. Where they course down the sides of the swelling they lose their central reflection or light streak, look darker at this point, and appear foreshortened, or as if hooking over the edge of the disc, or changed, or kinked in their course. The veins show this appearance most.

With these three essential conditions present, then, viz., change of color, obscuration of the outlines, and swelling of the disc, optic neuritis may be diagnosticated. But frequently other significant appearances are super-added. Thus, constriction of the vessels as they pass through the lamina cribrosa and the end of the optic nerve, narrows the retinal artery, and, in the eye, it and its branches become diminished in size. The outflow of blood through the retinal vein is, at the same time, impeded, and this vessel and its branches become engorged, and both their length and diameter are increased. The result is that the veins within the eye look tortuous, enlarged, and sometimes varicose. The stasis also leads to hemorrhages, here and there, in the disc and retina, more or less numerous and large. The swelling, also, more or less surrounds the vessels at the disc, according to its amount, and these may be hidden from view, especially the arteries, as they are more deeply situated and straighter, the tortuous veins showing themselves here and there at the points where they prominently curve forward. The inflammatory process may also throw out exudates on the disc, or just at its margin, or perhaps beyond, which have the appearance of whitish or flake-like spots or patches. The tortuous, distended, varicose-looking veins, the disappearance of the vessels here and there from sight, the radiating hemorrhages, and sometimes the white patches on or near the side of the disc, give, then, additional distinctiveness to the essential features of the disease, and all taken together present an impressive and unmistakable picture.

### SUBSIDENCE OF NEURITIS.

As the inflammation and edema subside, the disc returns to its normal condition, if the disease has been mild and connective-tissue proliferation slight. But more frequently the exudates become organized, contraction with constriction of the nerve-fibers and capillary vessels follow, and, by a process of degeneration, the optic nerve and retina pass into a state of atrophy. The first indication of subsidence is diminution of swelling. Then the vessels come more into view; the veins are less distended (although their tortuosity may continue long after the disease has passed away); they are less varicose; new hemorrhages cease to take place; old ones become absorbed, leaving behind white, or pigment spots, or no remains whatever; the disc gradually comes into view again, and it becomes clearly defined in outline, and, if the inflammation has been intense, it finally becomes very white.

Thus, with all possible intermediate types and results, the cycle of changes is wrought. The time required to complete such a cycle also varies greatly, even in cases exhibiting about the same type and form. It may take a few weeks, six to eight, or it may take as many months, or even a year, or more. The stage of onset is the shortest, the stage of persistence next longer, and that of subsidence the longest, although this is often shorter than the persisting stage.

### STATE OF VISION.

The only subjective symptom attending neuritis is impairment of vision, either in the field of vision or for objects and colors. But this is often out of all proportion to the appearance of the disc. In some well-marked and, to all appearances, severe cases, the vision seems to be very little, if at all, affected. On the other hand, it suffers very much while the disc-symptoms are less pronounced. Again, the vision may be very much impaired during the active stage, and return after the disease subsides. Or the vision may be greatly affected at first, improve as the neuritis diminishes, and again be lost during the processes of degeneration and atrophy.

### CAUSES OF NEURITIS.

With this brief outline of the characteristics which mark the progress of optic neuritis, let us now inquire, What causes may produce it?

In the first place, it may arise idiopathically, without any obvious cause, or from exposure to cold, disorders of menstruation, etc. Such cases are, however, very rare.

Secondly, it is sometimes caused by constitutional diseases, such as anemia, chlorosis, leucocythemia, scarlet fever, typhoid fever, measles, and by such diseases as diabetes mellitus, albuminuria, lead-poisoning, etc.

Thirdly, it may be due to intra-cranial disease, intra-cranial tumors producing by far the largest number of cases.

Fourthly, it may be caused by some disease within the orbit, such as cellulitis, orbital tumors, narrowing of the optic foramen by hyper-octures, etc.

Optic neuritis is, with rare exceptions, always double, unless the cause lays within the orbit. When monocular neuritis has been caused by brain tumor, it has occurred on the side opposite to that of the tumor.

Neuritis, as a rule, presents nothing which distinguishes its cause. An exception, however, may possibly be made when it is caused by albuminuria, or, perhaps, by one or two other diseases. But even then the exception could not apply, were it not for an association of changes in the retina which are distinctive of those causal diseases.

The physician determines the true cause of neuritis, first, by inference, attributing it to that affection, or class of affections, which most frequently produce it; second, by the associated symptoms and signs; third, by the history of the case. Now, the vast majority of cases are caused by some lesion of the brain, especially some growth or neoplasm. If he sees a case of neuritis, his first inference is that there is a brain tumor, or, if not that, some other brain disease. If the patient also has headache, vomiting, dizziness, convulsions, and other symptoms of brain tumor, the inference is probable. It is further strengthened if the history of the case shows syphilitic infection, or tubercular disease, or malignant disease, either in the patient or ancestors. The inference that the neuritis is due to brain tumor or brain disease, must, however, be changed if the accompanying symptoms, retinal changes, and history point to albuminuria, diabetes, lead poisoning, or other disease.

### PATHOGENESIS.

The interesting question, How is optic neuritis produced? claims a moment's consideration. Its pathogenesis in systemic diseases is unknown. Possibly, in some cases an irritant in the blood induces this localized inflammation. In others it may be brought about through consecutive disease of the brain or its meninges. Various theories have been advanced to explain the manner in which intra-cranial disease may produce the neuritis.

Three prominent conditions have been noticed in the pathological anatomy of neuritis: (1) an interstitial inflammation of the nerve, or in other words, an inflammation affecting the connective-tissue, or the inter-fasicular or lamellar structure of the nerve, which is derived from the pial sheath; (2) a distension of the inter-vaginal space with cerebro-spinal fluid, this distension being greatest very near the eye-ball; and (3) an edema of the optic disc.

From these and some other conditions have been derived and promulgated four hypotheses which, either singly, modified, or combined, are variously accepted to-day in explanation of this question:

*First.* That the neuritis is an inflammation descending from the brain in the nerve to the eye.

*Second.* That some vaso-motor or reflex influence from the brain induces the inflammation in the nerve and disc.

*Third.* That pressure within the optic-nerve sheath causes blood-stasis, or lymph-stasis, or both, and mechanically leads to neuritis. (Von Graefe advanced the theory of blood-stasis caused by intracranial pressure upon the cavernous sinus.)

*Fourth.* That the cerebro-spinal fluid is forced into the inter-vaginal space around the optic nerve, and produces not only distension, but carries with it pathogenic or infectious material, which causes inflammation by its entrance into the lymph-spaces of the nerve and disc.

The pathological, and, perhaps, the clinical appearances seem to point to more than one mechanism in producing this disease. That the inflammation does descend from the brain seems to account most rationally for those cases where there is no distension of the nerve-sheath. That distension of the sheath, and consequent compression of the bulbar end of the nerve, produces stasis of the outflowing blood- and lymph-currents from the eye, and results in edema and other changes of the disc, seems to be a reasonable explanation of those cases where the disc principally suffers, and the nerve-stem is very little inflamed.

Mr. R. Brudenell Carter, of London, has clinically reinforced and strengthened the present theory of "choked disc," whether the changes be brought about by stasis (as he holds), or by Deutschmann's infective material in the distending fluid. He has reported four cases of double "choked disc," with impaired vision, in which, by a special operation and special instrument, he has opened the optic-nerve sheath and drained the inter-vaginal space. The operation was performed only on the left eye, but the result was a rapid subsidence of the disease in both eyes, and a restoration of the vision, more or less complete, in each case. What greater proof could be offered of the truth that "choked disc" may be caused by the presence of fluid in the inter-vaginal space, which either produces compression around the optic-nerve entrance and stasis, or carries with it an infective irritant from the diseased brain and lights up the inflammation of the disc and nerve?

### SIGNIFICANCE OF NEURITIS AS A SYMPTOM.

So seldom is double optic neuritis a primary idiopathic affection, and so generally is it secondary to some other disease, that it may be practically regarded as a symptom. And so seldom, also, is it secondary to any but brain affections, that it may further be looked upon as a symptom of such affections. And limiting it still further, as it is in brain-tumors that it occurs in the great majority of cases, it becomes, therefore, in a somewhat qualified sense, a symptom of brain tumors. Furthermore, not only is neuritis due to intra-cranial tumor in such a large proportion of cases, but it is said to be present in at least four-fifths of those afflicted with such tumors.

Thus it has its greatest and most distinctive significance as a symptom of growths within the cranium, and as such is of great value. And yet it does not in any way indicate what the character ot the growth is,—whether it is malignant or benign, solid or cystic, small or large, old or recent, slow or rapid in its progress, ar aneurism or an abscess, or single or multiple. It may barely suggest the location of the growth, as it has been found to occur much more frequently when the lesion is in the cerebellum and basal ganglia, than in the convexity of the brain. It cannot, however, be considered in the light of a localizing or defining symptom. Like headache, vomiting, vertigo, convulsions, etc., it must be classed with them as a general symptom. But it greatly outweighs them, as such, in value. This becomes apparent when it is remembered that it is found only exceptionally in other diseases, and is present in a large proportion ot cases of brain tumor. Moreover, it is an objective sign, which can be recognized by the physician independently of the statements of the patient. In other words, it has a restricted significance, which the other symptoms do not possess. Thus, headache is an important symptom, but this, even persistent and severe, is often present in other diseases. Vomiting, without obvious cause, occurs in brain tumor, but it is found in a variety of other conditions. Vertigo is often present as a symptom, but there is nothing in it which distinguishes it from vertigo of aural or other disease. Convulsions, initiated by loss of consciousness, are another symptom, but they do not differ essentially from those of epilepsy. Mental disturbances also arise, but they are much more frequently due to other causes. Not so with double optic neuritis. This is seldom found outside of brain tumor. Hence it has a value in the diagnosis of this disease which is not possessed by any or all other symptoms,—a value which is too precious to be lost. With it, a certainty is given to the diagnosis; without it, there is much more uncertainty, and a large number and variety of other symptoms are necessary to remove the doubt. With that assurance which its presence with other general symptoms and history gives, and with the "focalizing" light which may be added by the so-called focal symptoms, such as local palsies, local spasms of various kinds, convulsions, beginning always at a certain part, affections of speech, local disturbances of sensation, hemianopsia, affections of taste, smelling, and hearing, etc., the physician is often enabled not only to arrive at a correct diagnosis, but he can determine the character of the growth, and give its precise location. Further, with such a result in diagnosis, he can confidently direct the proper treatment for the greatest relief of his patient, even to pointing out just where to cut to secure its removal, and thus making possible an operation which has become the greatest triumph of modern surgery.

212 Franklin Street.

